# Post-procedural access blood flow and repeat endovascular intervention in hemodialysis vascular access: a recurrent-event cohort study

**DOI:** 10.1080/0886022X.2026.2697399

**Published:** 2026-08-24

**Authors:** Hong-Mou Shih, Cheng-Jui Lin, Shih-Ming Chuang, Kuei-Shan Chang, Chih-Jen Wu, Chi-Feng Pan

**Affiliations:** ^a^ Division of Nephrology, Department of Internal Medicine, MacKay Memorial Hospital, Taipei, Taiwan; ^b^ School of Medicine, College of Medicine, MacKay Medical University, Taipei, Taiwan; ^c^ Graduate Institute of Physiology, College of Medicine, National Taiwan University, Taipei, Taiwan; ^d^ Nursing and Management, MacKay Junior College of Medicine, Taipei, Taiwan; ^e^ Division of Endocrinology and Metabolism, Department of Internal Medicine, MacKay Memorial Hospital, Taipei, Taiwan; ^f^ Department of Medical Research, China Medical University Hospital, China Medical University, Taichung, Taiwan

**Keywords:** Access blood flow, endovascular intervention, hemodialysis, stenosis

## Abstract

The prognostic value of access blood flow (Qa) measured after endovascular intervention is uncertain. We performed an episode-based recurrent-event cohort study of maintenance hemodialysis patients undergoing endovascular intervention for arteriovenous access dysfunction. The primary exposure was post-procedural Qa modeled continuously per 100 mL/min higher Qa; post-procedural Qa <1000 mL/min was examined as a secondary clinically interpretable threshold. The outcome was time to the next access intervention, analyzed using an Andersen–Gill counting-process Cox model with patient-level cluster-robust variance. A total of 131 patients contributed 382 intervention episodes, and post-procedural Qa was available for 315 episodes. Median time to repeat intervention was shorter for Qa <1000 mL/min than for Qa ≥1000 mL/min. In the primary adjusted model, each 100 mL/min higher post-procedural Qa was associated with a lower hazard of repeat intervention (aHR 0.941, 95% CI 0.896–0.990, *p* = 0.018). In the secondary threshold-based analysis, post-procedural Qa <1000 mL/min was associated with a higher hazard of repeat intervention (aHR 2.005, 95% CI 1.351–2.974, *p* < 0.001). Findings were generally consistent across sensitivity analyses, including IPW for missing post-procedural Qa, exclusion of occlusion episodes, shared-frailty modeling, adjustment for available pre-procedural Qa, and cause-specific terminal-event analysis. Lower post-procedural Qa may serve as a practical hemodynamic marker for post-procedural risk stratification after endovascular intervention.

## Introduction

Chronic kidney disease (CKD) is an expanding global health challenge, with a substantial and growing burden across regions and age groups. Recent global estimates indicate that CKD affects hundreds of millions worldwide and its prevalence continues to rise, underscoring its role as a major driver of morbidity and health-system demand [1]. A proportion of patients with progressive CKD ultimately develop end-stage kidney disease (ESKD) requiring kidney replacement therapy, most commonly maintenance hemodialysis (HD).

Despite advances in dialysis care, outcomes in maintenance HD remain poor, characterized by frequent hospitalizations and high mortality, with cardiovascular disease as the leading cause of adverse events [2,3]. For long-term survival, sustained delivery of adequate HD is essential, and this in turn depends on reliable vascular access, typically an arteriovenous (AV) fistula (AVF) or graft (AVG). Access-related complications are common and associated with substantial clinical burden, including hospitalizations, catheter dependence, interruptions in prescribed dialysis delivery, and recurrent endovascular procedures [4–6]. Among these, vascular access dysfunction is particularly common. Progressive stenosis is the predominant mechanism underlying vascular access dysfunction and may culminate in thrombosis and loss of access function, often necessitating urgent catheter placement and repeat procedures [5,7]. Consequently, vascular access–related morbidity represents a major driver of hospitalization and healthcare costs [8,9]. Accordingly, contemporary guidelines emphasize routine clinical monitoring to identify clinically significant access dysfunction and timely referral for diagnostic evaluation and targeted endovascular intervention, rather than preemptive angioplasty for stenosis in the absence of clinical indicators [10].

Percutaneous endovascular intervention, most commonly balloon angioplasty, has become a routine approach for access salvage. By dilating hemodynamically significant stenotic segments, angioplasty restores luminal caliber and access blood flow to preserve access usability and reduce thrombosis and access failure. In thrombosed accesses, endovascular thrombectomy is often performed to reestablish patency, followed by angioplasty of the underlying culprit stenosis. However, long-term outcomes after angioplasty-based interventions are suboptimal, and restenosis with repeat interventions remains common [11]. Accordingly, post-procedural risk stratification is clinically relevant. Objective hemodynamic markers may help identify episodes at risk for early recurrence.

Routine surveillance of access blood flow (Qa) is widely used to detect clinically important stenosis and to monitor access performance. Qa can be measured noninvasively and repeatedly, either by ultrasound dilution or Doppler-based approaches, providing a quantitative hemodynamic metric that complements bedside clinical monitoring [5]. Persistently low Qa may prompt closer evaluation, particularly when accompanied by clinical indicators of access dysfunction, consistent with guideline frameworks that prioritize intervention in the presence of clinical signs rather than preemptive angioplasty for stenosis alone [10]. Importantly, the magnitude of flow restoration after endovascular intervention varies across episodes, potentially reflecting residual stenosis, diffuse disease, or rapid recoil, and may therefore carry prognostic information [12].

Nevertheless, the prognostic value of Qa measured after endovascular intervention remains insufficiently defined, particularly in recurrent-intervention settings where patients may undergo repeated procedures over time. To address this gap, we examined the association between episode-specific post-procedural Qa, modeled primarily as a continuous hemodynamic marker, and repeat endovascular intervention using recurrent-event analyses. A threshold-based analysis using post-procedural Qa <1000 mL/min was additionally performed as a secondary, clinically interpretable analysis.

## Methods

### Study design and participants

We conducted a retrospective observational cohort study at MacKay Memorial Hospital, Taipei, Taiwan. Adults with ESKD receiving maintenance HD *via* an AVF or AVG who underwent endovascular intervention for vascular access dysfunction between January 2018 and December 2021 were eligible. Endovascular intervention was performed for clinically suspected AV access dysfunction based on routine clinical monitoring and access surveillance findings, including reduced dialysis adequacy, elevated venous pressure, difficult cannulation, prolonged bleeding after needle withdrawal, changes in thrill or bruit, arm swelling, and thrombosis or occlusion.

### Data collection and definitions

Clinical and laboratory data were retrieved from the electronic medical records and the dialysis information system. Variables collected included demographics (age and sex); access type (AVF or AVG) and location (left/right forearm or upper arm); comorbidities including diabetes mellitus (DM) and coronary artery disease (CAD); medication use (antiplatelet and anticoagulant therapy, angiotensin-converting enzyme inhibitor/angiotensin II receptor blocker, calcium channel blocker, and beta-blocker); pre-dialysis blood pressure (systolic and diastolic); and laboratory parameters including hemoglobin, serum albumin, total cholesterol, triglycerides, uric acid, pre-dialysis blood urea nitrogen, creatinine, sodium, potassium, calcium, phosphorus, and intact parathyroid hormone. CAD was defined as a documented history of myocardial infarction or angiographically confirmed CAD on cardiac catheterization. DM was defined by a prior clinical diagnosis with current or prior use of glucose-lowering medications and/or insulin therapy.

### Analytic unit, Qa measurement, and outcomes

The analytic unit was the endovascular intervention episode. Endovascular procedures consisted primarily of percutaneous transluminal angioplasty for stenotic lesions; in thrombosed or occluded accesses, endovascular thrombectomy followed by angioplasty of the underlying lesion was also performed. During the study period, each patient contributed a single AV access, while patients could contribute multiple intervention episodes over follow-up. A total of 382 intervention episodes were identified. Vascular access blood flow (Qa) was measured during HD using the ultrasound dilution technique with a Transonic HD03 monitor [13,14]. Measurements were performed within the first 30 min of the session after the blood pump speed had been stabilized. For each measurement episode, the blood pump speed was kept constant during all three consecutive readings and was required to be at least 200 mL/min. Qa was recorded as the mean of these three readings. Post-procedural Qa was scheduled within 7 days after each intervention; 67 episodes lacked a post-procedural Qa measurement within this 7-day window and were excluded from analyses requiring Qa. If more than one Qa measurement was available within this 7-day window, the earliest measurement was used.

The primary outcome was time from an intervention episode to the next access intervention, defined as repeat intervention. Follow-up time was calculated from the date of each intervention episode to the date of the subsequent repeat intervention. In the primary recurrent-event analysis, episodes without a subsequent repeat intervention were censored at the earliest of death, access abandonment, transfer to another hospital, administrative last follow-up, or the end of available follow-up. Because death and access abandonment could terminate follow-up for the access and preclude observation of subsequent repeat intervention, we further evaluated their potential impact using a separate cause-specific terminal-event sensitivity analysis.

## Statistical analysis

The primary exposure was post-procedural Qa modeled as a continuous variable per 100 mL/min higher Qa. A threshold-based analysis using post-procedural Qa <1000 mL/min was performed as a secondary clinically interpretable analysis. Prespecified covariates for multivariable adjustment included age, sex, access type, access location, DM, CAD, antiplatelet therapy, hemoglobin, serum albumin, and pre-dialysis systolic blood pressure. Because anticoagulant therapy was rare in this cohort, it was not included as a covariate in the primary multivariable model to avoid unstable estimates. Recurrent events were analyzed using a counting-process Cox proportional hazards model within the Andersen–Gill framework. Episode-specific post-procedural Qa was modeled as an episode-level covariate, and within-patient correlation arising from multiple endovascular interventions per patient was addressed using patient-level cluster-robust (sandwich) variance estimators. Multivariable models were fitted using complete-case data for the prespecified covariates.

Because missing post-procedural Qa may have been informative, the primary analysis was restricted to episodes with available Qa, and an inverse probability of measurement weighting (IPW) sensitivity analysis was performed. Stabilized inverse probability of measurement weights were estimated using logistic regression for the probability of having an available post-procedural Qa measurement. The weighting model included age, sex, access type, access location, DM, CAD, antiplatelet therapy, hemoglobin, serum albumin, pre-dialysis systolic blood pressure, occlusion/thrombosis at the episode under analysis, access age, previous intervention count, available pre-procedural Qa, and an indicator for missing pre-procedural Qa. Weighted recurrent-event Cox models were then fitted using the same covariates as the primary model. As an additional sensitivity analysis, stabilized weights were truncated at the 1st and 99th percentiles to reduce the influence of extreme weights.

To address access-type heterogeneity, analyses were stratified by access type, and interaction terms between post-procedural Qa and access type were tested. To account for recurrent-event ordering and unobserved within-patient heterogeneity, we fitted a Prentice–Williams–Peterson (PWP) total-time model stratified by recurrence order and a gamma shared-frailty Cox model with a patient-level frailty term. Additional sensitivity analyses included excluding occlusion episodes, examining alternative post-procedural Qa cutoffs of 800, 900, 1100, and 1200 mL/min as *post hoc* exploratory analyses, further adjusting for available pre-procedural Qa in the subset with non-missing pre-procedural flow measurements, and performing a cause-specific terminal-event analysis accounting for death and access abandonment.

Kaplan–Meier curves were used to describe reintervention-free probability from the study-period first endovascular intervention, stratified by the post-procedural Qa group (<1000 vs ≥1000 mL/min) measured within 1 week after the first procedure. Potential nonlinearity in the association between post-procedural Qa and repeat intervention risk was evaluated using restricted cubic splines.

Patient and episode selection, including the availability of post-procedural Qa and the analytic samples used for the primary and sensitivity analyses, is summarized in Figure S1. All analyses were performed using R version 4.5.3 (R Foundation for Statistical Computing, Vienna, Austria). Recurrent-event Cox models, PWP total-time models, gamma shared-frailty Cox models, IPW analyses, cause-specific terminal-event sensitivity analyses, and restricted cubic spline analyses were performed using the survival and splines packages. A two-sided P value <0.05 was considered statistically significant.

## Results

The cohort included 131 patients contributing 382 intervention episodes. Baseline characteristics at the first intervention episode during the study period are presented in Table 1; the mean age at baseline was 65.3 years. Most patients had an AVF, and the left forearm was the most common access location. Episode-level access characteristics and outcomes by post-procedural Qa group are summarized in Table 2. Post-procedural Qa was available for 315 of 382 episodes (82.5%), of which 214 episodes (67.9%) had post-procedural Qa <1000 mL/min and 101 episodes (32.1%) had post-procedural Qa ≥1000 mL/min. Access type distribution was similar between the two Qa groups, whereas access location differed significantly. Repeat intervention occurred more frequently in the <1000 mL/min group than in the ≥1000 mL/min group (72.0% vs 54.5%, *p* = 0.002). The median time to repeat intervention was also shorter in the <1000 mL/min group than in the ≥1000 mL/min group (109.5 days [IQR, 73.5–636.2] vs 275.0 days [IQR, 105.0–840.0], *p* < 0.001).

**Table 1.** Baseline clinical characteristics and laboratory data at first endovascular intervention (*N* = 131).

Characteristics	Value
Age, years	65.3 ± 12.8
Sex, N (%)	
Male	79 (60.3%)
Female	52 (39.7%)
Access type, N (%)	
AVF	95 (72.5%)
AVG	36 (27.5%)
Access location, N (%)	
Left forearm	64 (48.9%)
Left upper arm	25 (19.1%)
Right forearm	28 (21.4%)
Right upper arm	14 (10.7%)
DM, N (%)	51 (38.9%)
CAD, N (%)	53 (40.5%)
Antiplatelet therapy, N (%)	43 (32.8%)
Anticoagulant therapy, N (%)	4 (3.1%)
Pre-dialysis SBP (mmHg)	144 (129–158)
Hemoglobin (g/dL)	10.7 (10.2–11.3)
Albumin (g/dL)	4.1 (3.8–4.3)
Occlusion at first intervention episode	9 (6.9%)

Notes: Data are presented as the mean value ± standard deviation, N(%) or median (IQR). Missing data were minimal at the first-intervention baseline. Missing values remained only for albumin (*n* = 1); all other baseline characteristics were complete.

AVF: arteriovenous fistula; AVG: arteriovenous graft; DM: diabetes mellitus; CAD: coronary artery disease; SBP: systolic blood pressure.

**Table 2.** Episode-level access characteristics and outcomes by post-procedural Qa group (*N* = 315).

	Overall (*N* = 315)	Qa <1000 mL/min (*n* = 214)	Qa ≥1000 mL/min (*n* = 101)	P value
Access characteristics				
Access type, n (%)				0.522
AVF	189 (60.0)	131 (61.2)	58 (57.4)	
AVG	126 (40.0)	83 (38.8)	43 (42.6)	
Access location, n (%)				<0.001
Left forearm	151 (47.9)	107 (50.0)	44 (43.6)	
Left upper arm	59 (18.7)	25 (11.7)	34 (33.7)	
Right forearm	75 (23.8)	66 (30.8)	9 (8.9)	
Right upper arm	30 (9.5)	16 (7.5)	14 (13.9)	
Access age, days, median (IQR)	1061.0 (562.0–2426.0)	969.5 (540.3–2432.3)	1233.0 (604.0–2330.0)	0.515
Previous interventions before episode, median (IQR)	1 (0–3)	1 (0–3)	1 (0–3)	0.185
Outcomes				
Repeat interventions, n (%)	209 (66.3)	154 (72.0)	55 (54.5)	0.002
Follow-up to next intervention, days, median (IQR)	156.0 (88.5–733.5)	109.5 (73.5–636.2)	275.0 (105.0–840.0)	<0.001

Notes: Values are presented as median (IQR) or n (%). Post-procedural Qa was missing in 67 of 382 episodes (17.5%). P values were calculated using the Mann–Whitney U test for continuous or ordinal variables and Pearson chi-square or Fisher’s exact test for categorical variables, as appropriate. Previous interventions before episode were defined as the number of prior intervention episodes before the episode under analysis.

Qa: access blood flow; IQR: interquartile range; AVF: arteriovenous fistula; AVG: arteriovenous graft.

**Figure 1.**
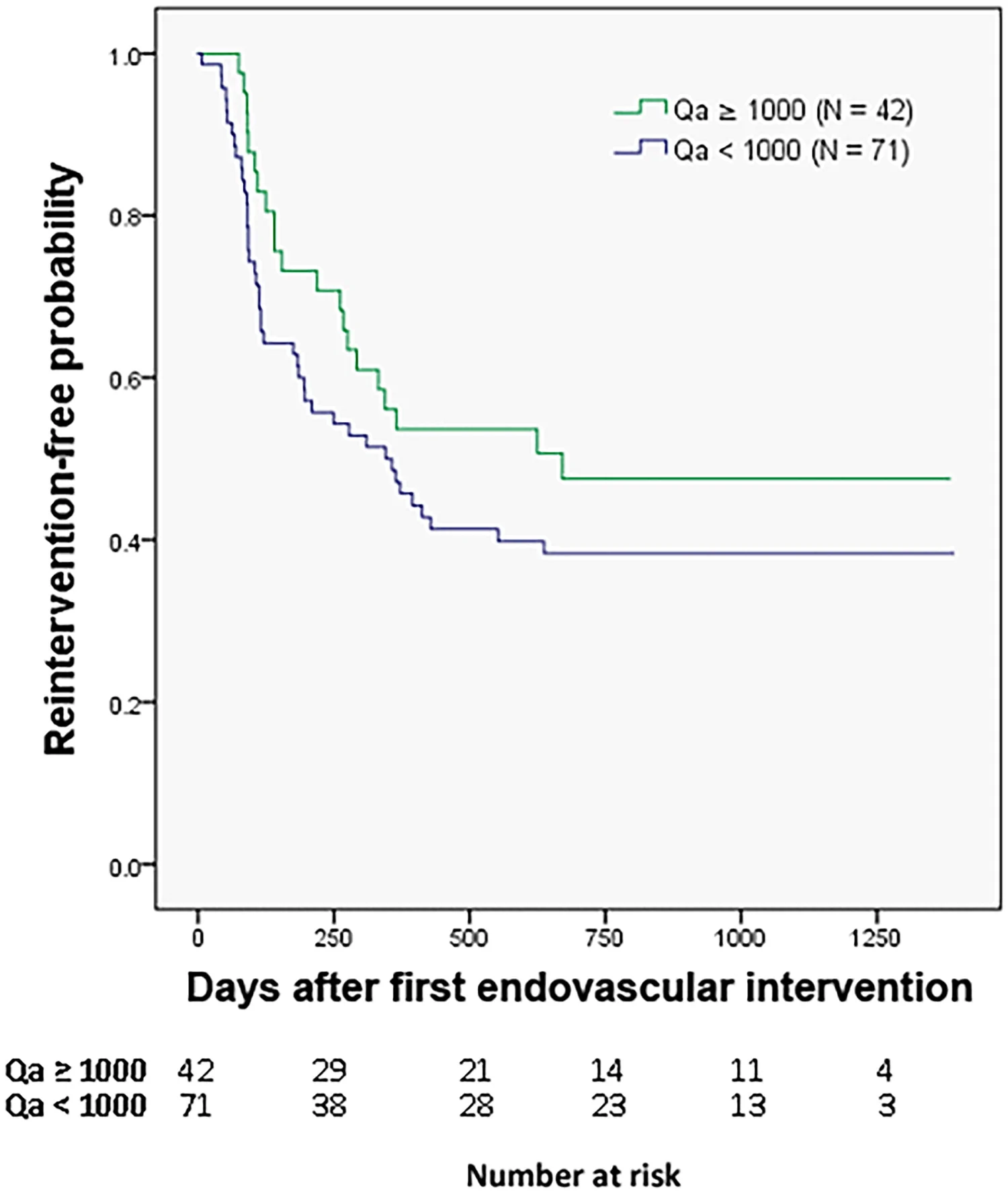
Kaplan–Meier curves for time to repeat intervention after the study-period first endovascular intervention, stratified by post-procedural Qa <1000 vs ≥1000 mL/min. This patient-level analysis included patients with available post-procedural Qa measured within 7 days after the first endovascular intervention during the study period (*N* = 113; Qa <1000 mL/min, *n* = 71; Qa ≥1000 mL/min, *n* = 42). Numbers at risk are shown below the curve.

**Figure 2.**
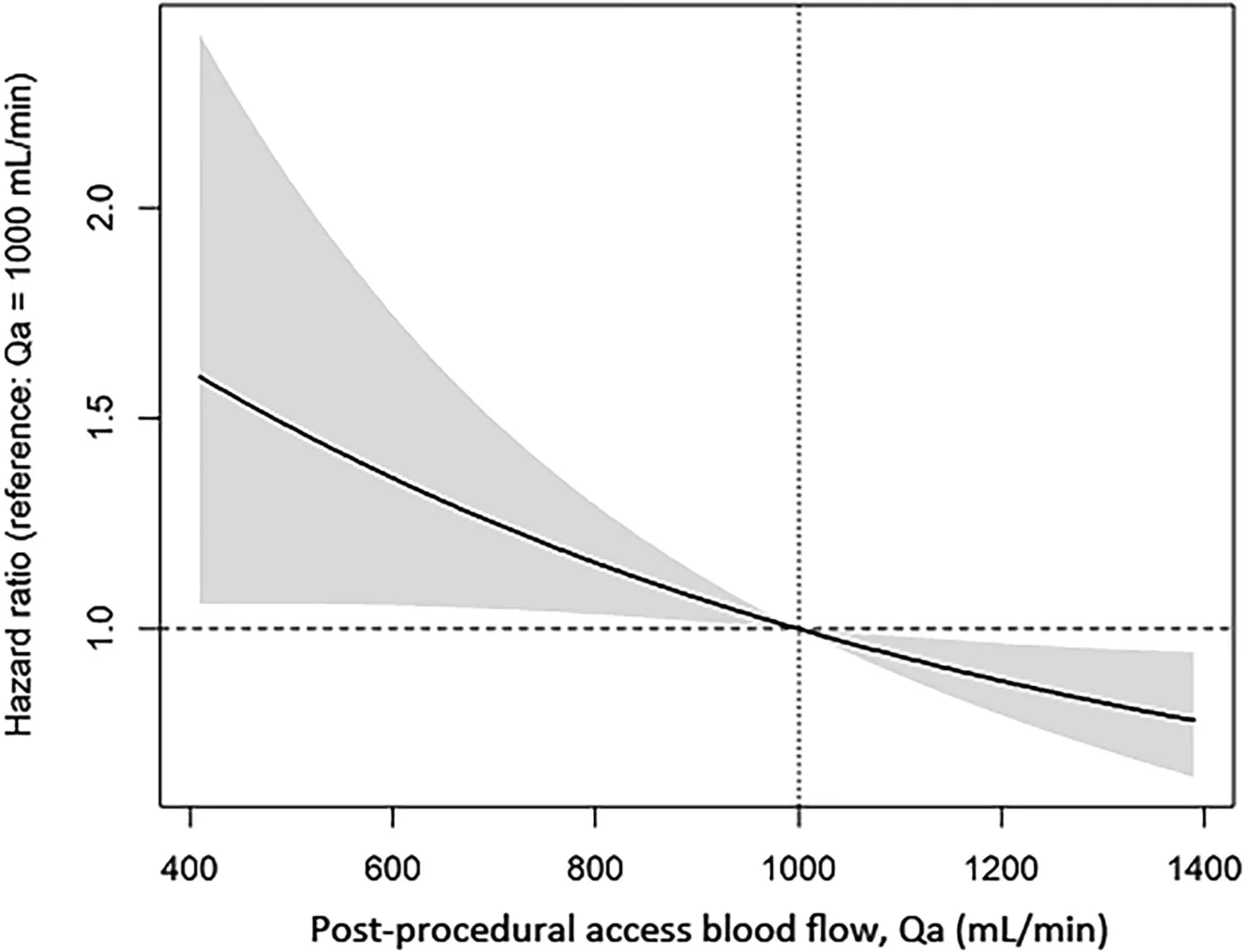
Restricted cubic spline showing the association between post-procedural access blood flow (Qa) and risk of repeat intervention. Note: Hazard ratios are shown relative to Qa = 1000 mL/min; the shaded area indicates the 95% confidence interval. The x-axis is displayed over the 10th–90th percentile range of post-procedural Qa.

In the primary recurrent-event model treating post-procedural Qa as a continuous variable, each 100 mL/min higher post-procedural Qa was associated with a lower hazard of repeat intervention after multivariable adjustment (adjusted hazard ratio [aHR] 0.941, 95% CI, 0.896–0.990, *p* = 0.018; Table 3). In a secondary threshold-based analysis, post-procedural Qa <1000 mL/min was associated with a higher hazard of repeat intervention (aHR 2.005, 95% CI 1.351–2.974, *p* < 0.001; Table S1). In patient-level Kaplan–Meier analysis, time zero was defined as the first endovascular intervention episode during the study period; reintervention-free probability appeared lower in patients with post-procedural Qa <1000 mL/min than in those with ≥1000 mL/min (Figure 1), although the between-group difference was not statistically significant by log-rank testing. Results were further supported by restricted cubic spline analysis, which evaluated the functional form of the association between post-procedural Qa and repeat intervention risk (Figure 2). Alternative threshold analyses using cutoffs of 800, 900, 1100, and 1200 mL/min showed generally directionally consistent associations, although the estimate was attenuated and did not reach statistical significance at the 1200 mL/min cutoff (Table S1). Sensitivity analysis excluding occlusion episodes also yielded similar results (Table S2).

**Table 3.** Primary adjusted recurrent-event cox model treating post-procedural Qa as a continuous variable for repeat intervention.

Covariate	Adjusted HR (95% CI)	P value
Post-procedural Qa, per 100 mL/min higher	0.941 (0.896–0.990)	0.018
Male sex	0.938 (0.613–1.435)	0.768
AVF (vs AVG)	0.633 (0.401–1.000)	0.050
DM	1.151 (0.799–1.660)	0.450
CAD	0.741 (0.513–1.069)	0.109
Antiplatelet therapy	1.385 (0.951–2.017)	0.090
Hemoglobin (per 1 g/dL)	1.064 (0.930–1.218)	0.369
Albumin (per 1 g/dL)	1.194 (0.710–2.008)	0.504
Access location (vs left forearm)		
Left upper arm	1.045 (0.647–1.686)	0.858
Right forearm	1.007 (0.664–1.527)	0.973
Right upper arm	1.031 (0.577–1.844)	0.918
Age (per 10 years)	0.937 (0.740–1.186)	0.587
Pre-dialysis SBP (per 10 mmHg)	1.027 (0.940–1.122)	0.557

Notes: Adjusted for age, sex, access type, access location, DM, CAD, antiplatelet therapy, hemoglobin, serum albumin, and pre-dialysis SBP using an Andersen–Gill recurrent-event Cox model with patient-level cluster-robust (sandwich) standard errors.

Qa: access blood flow; HR: hazard ratio; CI: confidence interval; AVF: arteriovenous fistula; AVG: arteriovenous graft; DM: diabetes mellitus; CAD: coronary artery disease; SBP: systolic blood pressure.

Access-type stratified and interaction analyses are shown in Table S3. In the primary continuous-Qa model, higher post-procedural Qa was associated with a lower hazard of repeat intervention among AVF episodes (aHR per 100 mL/min higher Qa, 0.907; 95% CI, 0.846–0.973; *p* = 0.006). Among AVG episodes, the association was directionally similar but did not reach statistical significance (aHR, 0.950; 95% CI, 0.875–1.031; *p* = 0.221). The interaction between post-procedural Qa and access type was not statistically significant (P for interaction = 0.918), indicating no clear evidence that the association between post-procedural Qa and repeat intervention differed by access type. In the secondary threshold-based analysis, post-procedural Qa <1000 mL/min was associated with a higher hazard of repeat intervention in both AVF episodes (aHR, 2.600; 95% CI, 1.530–4.410; *p* = 0.001) and AVG episodes (aHR, 1.850; 95% CI, 1.040–3.290; *p* = 0.036), with no significant interaction by access type (P for interaction = 0.858).

Because missing post-procedural Qa may have been informative, we performed an IPW sensitivity analysis (Table S4). In the continuous-Qa model, the association between higher post-procedural Qa and lower repeat-intervention risk remained essentially unchanged after weighting for the probability of having post-procedural Qa measured. The aHR per 100 mL/min higher post-procedural Qa was 0.941 (95% CI, 0.896–0.990; *p* = 0.018) in the unweighted complete-case model, 0.940 (95% CI, 0.893–0.989; *p* = 0.017) after IPW, and 0.939 (95% CI, 0.892–0.988; *p* = 0.016) after weight truncation. Similar findings were observed in the secondary threshold-based analysis: the aHR for post-procedural Qa <1000 mL/min was 2.005 (95% CI, 1.351–2.974; *p* < 0.001) in the unweighted complete-case model, 2.025 (95% CI, 1.365–2.944; *p* < 0.001) after IPW, and 2.038 (95% CI, 1.382–3.005; *p* < 0.001) after weight truncation.

In a Prentice–Williams–Peterson (PWP) total-time model stratified by recurrence order, the association between higher post-procedural Qa and lower repeat-intervention risk was directionally consistent but attenuated and did not reach conventional statistical significance (aHR per 100 mL/min higher Qa, 0.960; 95% CI, 0.916–1.005; *p* = 0.083). In the secondary threshold-based PWP model, post-procedural Qa <1000 mL/min remained associated with a higher hazard of repeat intervention (aHR, 1.740; 95% CI, 1.097–2.758; *p* = 0.018; Table S5).

In a gamma shared-frailty Cox sensitivity model with a patient-level frailty term, the association between higher post-procedural Qa and lower repeat-intervention risk remained consistent (aHR per 100 mL/min higher Qa, 0.934; 95% CI, 0.897–0.972; *p* = 0.001). The secondary threshold-based analysis using Qa <1000 mL/min also remained consistent in the frailty model (aHR, 2.060; 95% CI, 1.470–2.890; *p* < 0.001; Table S6).

In the subset of intervention episodes with available pre-procedural access flow measurements, further adjustment for pre-procedural flow did not materially alter the association between post-procedural Qa and repeat intervention. In the primary continuous model, higher post-procedural Qa remained associated with a lower hazard of repeat intervention after adjustment for pre-procedural flow (aHR per 100 mL/min higher Qa, 0.915; 95% CI, 0.853–0.981; *p* = 0.013; Table S7). The secondary threshold-based model was also consistent, with post-procedural Qa <1000 mL/min associated with a higher hazard of repeat intervention (aHR, 2.545; 95% CI, 1.484–4.367; *p* < 0.001).

In the cause-specific recurrent-event sensitivity analysis accounting for death and access abandonment as terminal competing events, the association between higher post-procedural Qa and lower repeat-intervention risk remained consistent with the primary analysis (aHR per 100 mL/min higher Qa, 0.934; 95% CI, 0.886–0.985; *p* = 0.011). The secondary threshold-based model using post-procedural Qa <1000 mL/min also yielded similar findings (aHR, 2.065; 95% CI, 1.427–2.987; *p* < 0.001; Table S8).

Overall, the sensitivity analyses supported the consistency of the association between post-procedural Qa and repeat-intervention risk.

## Discussion

In this episode-based recurrent-event study, lower post-procedural access blood flow was associated with a shorter interval to repeat endovascular intervention. When modeled continuously, higher post-procedural Qa was associated with a lower hazard of repeat intervention, and the secondary threshold-based analysis showed that post-procedural Qa <1000 mL/min measured within 7 days after each intervention episode was associated with a higher hazard of subsequent intervention after multivariable adjustment. These findings suggest that the early hemodynamic response after endovascular intervention may capture the combined effects of the immediate procedural result and the underlying access condition. Low post-procedural Qa should therefore not be interpreted as a lesion-independent or procedure-independent causal factor. Rather, it may reflect residual stenosis, incomplete lesion dilation, diffuse or multifocal disease, elastic recoil, limited flow reserve, early restenosis, or other unmeasured lesion- and procedure-related factors. Accordingly, the observed association is best interpreted as a post-procedural risk-stratification signal rather than evidence that low Qa directly causes repeat intervention. The clinically interpretable threshold of post-procedural Qa <1000 mL/min provided meaningful risk stratification, and the association remained consistent after excluding occlusion episodes.

Observational and prospective studies have linked access blood flow to vascular access prognosis and patient survival, supporting Qa as a physiologically meaningful marker of access performance. In incident HD patients, early vascular access blood flow has been associated with subsequent long-term access patency [15]. Similarly, Qa measured at the time of first cannulation has been related to subsequent vascular access outcomes in maintenance HD [16]. In native AV fistulas, Qa-based screening has been used to detect subclinical stenosis and identify accesses at higher risk of dysfunction [17]. Moreover, prospective studies evaluating multiple indicators of access deterioration have identified Qa as an important predictor of subsequent access thrombosis [18]. In addition to access-related endpoints, lower Qa has also been associated with higher short-term and long-term mortality in a chronic HD cohort, suggesting that Qa may correlate with overall patient risk [19]. Collectively, these data provide a rationale for evaluating Qa as part of hemodynamic risk assessment.

However, whether routine Qa surveillance programs improve outcomes beyond clinical monitoring remains uncertain. An ultrasound-dilution–based flow-monitoring program coupled with preemptive evaluation and intervention for suspected stenosis was associated with reductions in thrombosis-related morbidity, including fewer hospital days, fewer missed treatments, and fewer catheter placements, as well as lower overall costs, despite an increase in angioplasty procedures [9]. A prospective observational study similarly suggested that flow-based surveillance can identify clinically significant stenosis and guide downstream evaluation and intervention [20]. In contrast, a comparative cohort analysis reported that standardized clinical assessment performed similarly to ultrasound flow surveillance with respect to access patency and rates of subsequent angioplasty [21]. Moreover, randomized trials in AV grafts found that adding Qa- or duplex-based stenosis surveillance increased the frequency of preemptive angioplasty but did not clearly reduce thrombosis rates or improve graft survival compared with clinical monitoring alone [22,23]. Taken together, Qa provides clinically meaningful hemodynamic information, but the additional benefit of routine surveillance appears context-dependent and may vary by access type and by how surveillance findings are incorporated into diagnostic evaluation and subsequent intervention. This heterogeneity of evidence is reflected in contemporary guideline recommendations. The 2019 KDOQI vascular access guideline emphasizes routine clinical monitoring and does not recommend preemptive angioplasty for stenosis in the absence of clinical indicators [5]. Therefore, our study focused on post-procedural risk stratification using episode-specific post-procedural Qa to help inform risk-based follow-up after the procedure, rather than advocating preemptive intervention in the absence of clinical indicators.

Several studies have evaluated post-angioplasty access flow as a functional measure of procedural response and a potential prognostic marker. Van der Linden and colleagues showed that although endovascular intervention improves stenosis angiographically, the degree of stenosis reduction correlates poorly with access outcomes; in contrast, baseline Qa and the post-procedural rise in Qa (ΔQa) better predict subsequent access performance [24]. Additional hemodynamic studies, using Doppler-derived parameters or examining determinants of post-procedural flow change, further suggest that early flow responses vary across intervention episodes and may carry clinically relevant information [25–27].

However, the existing literature has primarily focused on access-level patency endpoints, and post-procedural flow has been less frequently examined on an episode-by-episode basis using methods that incorporate repeated intervention episodes within patients. Accordingly, in a cohort comprising both AVFs and AVGs, we evaluated post-procedural Qa after each endovascular intervention episode using recurrent-event methods to reflect real-world patterns of repeated intervention over time. Our findings are consistent with prior evidence supporting the prognostic value of post-procedural access flow. For example, in AVGs, Murray et al. reported that higher post-procedural flow (with thresholds around 1000 mL/min) was associated with fewer repeat interventions and longer assisted patency [12]. Extending these observations to an episode-based framework across AVF and AVG access types, our study suggests that a clinically interpretable threshold may be useful for post-procedural risk stratification and may help inform risk-based follow-up in this cohort.

Our access-type stratified analyses further address the concern that AVFs and AVGs may have different baseline flow distributions and distinct hemodynamic behavior. In the primary continuous-Qa model, the association between higher post-procedural Qa and lower repeat-intervention risk was statistically significant among AVF episodes and directionally similar among AVG episodes. The secondary threshold-based analysis also showed that post-procedural Qa <1000 mL/min was associated with a higher hazard of repeat intervention in both access types. Importantly, formal interaction testing did not show statistically significant effect modification by access type. These findings suggest that the prognostic information carried by post-procedural Qa was not clearly restricted to either AVFs or AVGs. Nevertheless, the AVG subgroup was smaller, and the absence of a statistically significant interaction should not be interpreted as definitive evidence that the magnitude of association or the optimal Qa threshold is identical across access types.

Several sensitivity analyses further supported the robustness of our findings. The association between post-procedural Qa and repeat intervention remained consistent after excluding occlusion episodes and across alternative threshold-based analyses using cutoffs of 800, 900, 1000, 1100, and 1200 mL/min, although the 1200 mL/min cutoff did not reach statistical significance. The association was directionally consistent in a PWP total-time model stratified by recurrence order and remained consistent in a gamma shared-frailty Cox model with a patient-level frailty term, suggesting that the main findings were not solely dependent on the Andersen–Gill modeling framework. In the subset with available pre-procedural access flow measurements, further adjustment for pre-procedural flow did not materially alter the association between low post-procedural Qa and repeat intervention. Similar findings were also observed in the cause-specific recurrent-event sensitivity analysis accounting for death and access abandonment as terminal competing events. Taken together, these findings suggest that post-procedural Qa may serve as a practical risk-stratification marker after endovascular intervention. However, the 1000 mL/min threshold should not be interpreted as a universally validated cutoff applicable across all access types or populations. Post-procedural Qa should therefore be interpreted in the broader clinical context rather than as a standalone target to be maximized.

Missing post-procedural Qa deserves careful consideration because its absence may reflect clinical instability, access thrombosis, logistical constraints, or other episode-level features that are also related to subsequent access outcomes. Therefore, we did not assume that missing Qa was completely random. Instead, we performed an IPW sensitivity analysis to account for the probability of having post-procedural Qa measured. The primary association between higher post-procedural Qa and lower repeat-intervention risk remained essentially unchanged after weighting, and the results were also stable after weight truncation. These findings support the robustness of the primary analysis to potential informative missingness in post-procedural Qa. Nevertheless, IPW can only account for measured factors associated with Qa availability, and residual bias from unmeasured reasons for missing Qa cannot be excluded.

The difference between the Kaplan–Meier and recurrent-event analyses should be interpreted in light of their different analytic targets. The Kaplan–Meier analysis was performed at the patient level from the study-period first intervention episode and therefore summarized time to the first subsequent intervention only. In contrast, the Andersen–Gill recurrent-event models incorporated all eligible intervention episodes and accounted for within-patient correlation, providing a more appropriate framework for evaluating repeated access interventions over time.

The imbalance in access location between post-procedural Qa groups also warrants consideration. Access location may influence achievable flow, lesion distribution, cannulation history, and subsequent intervention risk. Although access location was adjusted for in the multivariable models, residual confounding related to access anatomy and lesion characteristics cannot be fully excluded. This further supports interpreting post-procedural Qa as an integrated hemodynamic risk marker rather than a lesion- or procedure-independent predictor.

These findings support the potential use of post-procedural Qa for episode-level risk stratification and provide a rationale for tailoring post-procedural surveillance. Clinically, an episode-specific post-procedural Qa threshold may help inform the intensity and timing of follow-up after the procedure, although caution is needed when applying a single Qa threshold across different access types, given the differing baseline flow characteristics of AVFs and AVGs. Episodes with low post-procedural Qa may merit closer surveillance, with earlier diagnostic evaluation guided by evolving clinical indicators such as changes in the access thrill or bruit, arm swelling, or prolonged post-dialysis bleeding [5]. Reinforcing patient education on routine access self-examination, including daily palpation of the access thrill and prompt reporting of any change, may further complement unit-based monitoring and facilitate earlier recognition of access compromise. Future studies should validate optimal post-procedural-Qa relates to lesion characteristics and procedural factors, and determine whether Qa-informed follow-up strategies, potentially combined with carefully selected adjunctive therapies, can reduce early recurrence without unnecessary interventions.

Strengths of this study include detailed episode-level characterization and the use of recurrent-event modeling with cluster-robust variance to account for repeated episodes per patient. Several limitations should also be acknowledged. First, this was a retrospective single-center study, and post-procedural Qa was missing in a subset of episodes. Although IPW was used to address potentially informative missingness, residual bias from unmeasured reasons for missing Qa cannot be excluded. Second, Qa measurements may be subject to measurement variability despite standardized measurement procedures. Third, several potentially important lesion- and procedure-level factors were not consistently available, including stenosis severity, residual stenosis after intervention, lesion number and location, balloon type, drug-coated balloon use, and stent placement. These factors may influence restenosis, repeat intervention risk, and post-procedural Qa. Therefore, low post-procedural Qa may partly reflect unmeasured lesion complexity or procedural response rather than an independent causal effect. This limitation reinforces that post-procedural Qa should be regarded as an integrated hemodynamic risk marker to inform follow-up intensity, not as a standalone target or replacement for angiographic and clinical assessment. Fourth, recurrent intervention counts were unevenly distributed across patients, with a subset of patients contributing multiple repeat interventions (Figure S2). Although the primary recurrent-event analysis used the Andersen–Gill model with patient-level cluster-robust variance estimators, residual event dependence and subject-specific heterogeneity cannot be fully excluded. However, the association remained directionally consistent in a PWP total-time model stratified by recurrence order (Table S5), supporting the robustness of the main findings across recurrent-event modeling approaches. Finally, because standardized Qa measurements obtained under stable, non-stenotic conditions were not uniformly available, we could not determine the extent to which post-procedural Qa reflected the immediate procedural result versus underlying access-specific baseline hemodynamics.

In conclusion, lower post-procedural Qa was associated with earlier repeat endovascular intervention in this episode-based recurrent-event cohort. A clinically interpretable threshold of post-procedural Qa <1000 mL/min identified episodes at increased risk, while requiring validation across access types and populations. Post-procedural Qa may serve as a practical hemodynamic marker for post-procedural risk stratification and targeted follow-up after endovascular intervention.

## Supplementary Material

Supplemental Material

Supplemental Material

Supplemental Material

## Data Availability

The datasets analyzed during the current study are available from the corresponding author upon reasonable request.
