# Supplementary material for: Post-procedural access blood flow and repeat endovascular intervention in hemodialysis vascular access: a recurrent-event cohort study
Source: Ren Fail. 2026 Aug 24;48(1):2697399. doi: 10.1080/0886022X.2026.2697399 (PMC13508538; doi:10.1080/0886022X.2026.2697399)
Supplement: Supplementary file 1 [file IRNF_A_2697399_SM7676.docx]

| **Supplementary Table S1. Secondary threshold-based recurrent-event Cox analyses using alternative post-procedural Qa cutoffs** | | | |
| --- | --- | --- | --- |
| Qa cut-off | aHR | 95% CI | P value |
| 800 | 1.778 | 1.205–2.623 | 0.004 |
| 900 | 1.872 | 1.333–2.630 | <0.001 |
| 1000 | 2.005 | 1.351–2.974 | <0.001 |
| 1100 | 1.812 | 1.216–2.699 | 0.003 |
| 1200 | 1.462 | 0.963–2.220 | 0.075 |
| **Notes:** Adjusted for the same covariates as Table 3. Robust sandwich standard errors were computed by clustering on patient ID. All threshold-based analyses were considered secondary and exploratory. The 1000 mL/min cutoff was prespecified as a clinically interpretable threshold, whereas other cutoffs were examined as post hoc exploratory sensitivity analyses.  **Abbreviations:** Qa, access blood flow; aHR, adjusted hazard ratio; CI, confidence interval. | | | |

| **Supplementary Table S2. Sensitivity analysis after excluding occlusion episodes** | | |
| --- | --- | --- |
| Model | aHR (95% CI) | P value |
| Post-procedural Qa, per 100 mL/min higher | 0.938 (0.891–0.988) | 0.015 |
| Post-procedural Qa <1000 mL/min vs ≥1000 | 2.048 (1.366–3.071) | <0.001 |
| **Notes:** Adjusted for the same covariates as Table 3 using an Andersen–Gill recurrent-event Cox model with patient-level cluster-robust (sandwich) standard errors. **Abbreviations:** Qa, access blood flow; aHR, adjusted hazard ratio; CI, confidence interval. | | |

| **Supplementary Table S3. Stratified and interaction analyses by access type** | | | | |
| --- | --- | --- | --- | --- |
| Analysis | Access type | aHR (95% CI) | P value | P for interaction |
| Post-procedural Qa per 100 mL/min higher | AVF | 0.907 (0.846–0.973) | 0.006 | 0.918 |
|  | AVG | 0.950 (0.875–1.031) | 0.221 |  |
| Qa <1000 vs ≥1000 mL/min | AVF | 2.600 (1.530–4.410) | 0.001 | 0.858 |
|  | AVG | 1.850 (1.040–3.290) | 0.036 |  |
| **Notes:** Models were adjusted for the same covariates as Table 3. The primary analysis modeled post-procedural Qa as a continuous variable per 100 mL/min higher Qa. Interaction P values were obtained from product terms between post-procedural Qa and access type in the full cohort. **Abbreviations:** Qa, access blood flow; aHR, adjusted hazard ratio; CI, confidence interval. | | | | |

| **Supplementary Table S4. Inverse probability weighted sensitivity analysis for missing post-procedural Qa** | | | |
| --- | --- | --- | --- |
| Model | Analysis | aHR (95% CI) | P value |
| Post-procedural Qa, per 100 mL/min higher | Unweighted complete-case | 0.941 (0.896–0.990) | 0.018 |
|  | IPW | 0.940 (0.893–0.989) | 0.017 |
|  | IPW, truncated weights | 0.939 (0.892–0.988) | 0.016 |
| Post-procedural Qa <1000 mL/min vs ≥1000 | Unweighted complete-case | 2.005 (1.351–2.974) | <0.001 |
|  | IPW | 2.025 (1.365–2.944) | <0.001 |
|  | IPW, truncated weights | 2.038 (1.382–3.005) | <0.001 |
| **Notes:** Stabilized inverse probability weights were estimated from a logistic regression model for having an available post-procedural Qa measurement. The model included age, sex, access type, access location, diabetes mellitus, coronary artery disease, antiplatelet therapy, hemoglobin, serum albumin, pre-dialysis systolic blood pressure, index occlusion/thrombosis, access age, previous intervention count, available pre-procedural Qa, and an indicator for missing pre-procedural Qa. The weighted recurrent-event Cox model used the same covariates as Table 3. Stabilized weights were truncated at the 1st and 99th percentiles in the truncated-weight sensitivity analysis. **Abbreviations:** Qa, access blood flow; aHR, adjusted hazard ratio; CI, confidence interval; IPW, Inverse probability weighting. | | | |

| **Supplementary Table S5. Sensitivity analysis using a Prentice**–**Williams**–**Peterson total-time model stratified by recurrence order** | | | |
| --- | --- | --- | --- |
| Model | aHR | 95% CI | P value |
| Post-procedural Qa, per 100 mL/min higher | 0.960 | 0.916–1.005 | 0.083 |
| Post-procedural Qa <1000 mL/min vs ≥1000 | 1.740 | 1.097–2.758 | 0.018 |
| **Notes:** The PWP total-time model was stratified by recurrence order and adjusted for the same covariates as Table 3. Robust sandwich standard errors were clustered by patient ID.  **Abbreviations:** Qa, access blood flow; aHR, adjusted hazard ratio; CI, confidence interval; PWP, Prentice–Williams–Peterson. | | | |

| **Supplementary Table S6. Shared-frailty recurrent-event Cox sensitivity analysis** | | | |
| --- | --- | --- | --- |
| Analysis | Model | aHR (95% CI) | P value |
| Post-procedural Qa, per 100 mL/min higher | Cluster-robust recurrent Cox | 0.941 (0.896–0.990) | 0.018 |
|  | Gamma shared-frailty Cox | 0.934 (0.897–0.972) | 0.001 |
| Post-procedural Qa <1000 mL/min vs ≥1000 | Cluster-robust recurrent Cox | 2.005 (1.351–2.974) | <0.001 |
|  | Gamma shared-frailty Cox | 2.060 (1.470–2.890) | <0.001 |
| **Notes:** The gamma shared-frailty model included a patient-level frailty term to account for unobserved heterogeneity in baseline recurrent-intervention risk across patients. Models were adjusted for the same covariates as Table 3.  **Abbreviations:** Qa, access blood flow; aHR, adjusted hazard ratio; CI, confidence interval. | | | |

| **Supplementary Table S7. Sensitivity analysis additionally adjusting for available pre-procedural Qa** | | | |
| --- | --- | --- | --- |
| Model | aHR | 95% CI | P value |
| Post-procedural Qa, per 100 mL/min higher | 0.915 | 0.853–0.981 | 0.013 |
| Post-procedural Qa <1000 mL/min vs ≥1000 | 2.545 | 1.484–4.367 | <0.001 |
| **Notes:** Analyses were restricted to episodes with available pre-procedural Qa measurements, including 227 episodes from 100 patients. Models were additionally adjusted for pre-procedural Qa, in addition to age, sex, access type, access location, diabetes mellitus, coronary artery disease, antiplatelet therapy, hemoglobin, serum albumin, and pre-dialysis systolic blood pressure**.**  **Abbreviations:** Qa, access blood flow; aHR, adjusted hazard ratio; CI, confidence interval. | | | |

| **Supplementary Table S8. Cause-specific recurrent-event sensitivity analysis accounting for death and access abandonment as terminal competing events** | | | |
| --- | --- | --- | --- |
| Analysis | Model | aHR (95% CI) | P value |
| Post-procedural Qa, per 100 mL/min higher | Standard recurrent Cox model | 0.941 (0.896–0.990) | 0.018 |
|  | Cause-specific terminal-event model | 0.934 (0.886–0.985) | 0.011 |
| Post-procedural Qa <1000 mL/min vs ≥1000 | Standard recurrent Cox model | 2.005 (1.351–2.974) | <0.001 |
|  | Cause-specific terminal-event model | 2.065 (1.427–2.987) | <0.001 |
| **Notes:** Cause-specific terminal-event models recalculated follow-up for each intervention episode from each procedure episode under analysis to the earliest of repeat intervention, death, access abandonment, administrative transfer/last follow-up, or the end of available follow-up. Death and access abandonment were treated as terminal competing events. Models were adjusted for the same covariates as Table 3. Robust sandwich standard errors were computed by clustering on patient ID.  **Abbreviations:** Qa, access blood flow; aHR, adjusted hazard ratio; CI, confidence interval. | | | |
